# Paper Versus Digital Data Collection Methods for Road Safety Observations: Comparative Efficiency Analysis of Cost, Timeliness, Reliability, and Results

**DOI:** 10.2196/17129

**Published:** 2020-05-22

**Authors:** Niloufer Taber, Amber Mehmood, Perumal Vedagiri, Shivam Gupta, Rachel Pinto, Abdulgafoor M Bachani

**Affiliations:** 1 International Injury Research Unit Johns Hopkins Bloomberg School of Public Health Baltimore, MD United States; 2 Department of Civil Engineering Transportation Systems Engineering Indian Institute of Technology Bombay Mumbai India

**Keywords:** information technology, public health informatics, mHealth, risk factors, population surveillance, traffic accidents, data collection

## Abstract

**Background:**

Roadside observational studies play a fundamental role in designing evidence-informed strategies to address the pressing global health problem of road traffic injuries. Paper-based data collection has been the standard method for such studies, although digital methods are gaining popularity in all types of primary data collection.

**Objective:**

This study aims to understand the reliability, productivity, and efficiency of paper vs digital data collection based on three different road user behaviors: helmet use, seatbelt use, and speeding. It also aims to understand the cost and time efficiency of each method and to evaluate potential trade-offs among reliability, productivity, and efficiency.

**Methods:**

A total of 150 observational sessions were conducted simultaneously for each risk factor in Mumbai, India, across two rounds of data collection. We matched the simultaneous digital and paper observation periods by date, time, and location, and compared the reliability by subgroups and the productivity using Pearson correlations (r). We also conducted logistic regressions separately by method to understand how similar results of inferential analyses would be. The time to complete an observation and the time to obtain a complete dataset were also compared, as were the total costs in US dollars for fieldwork, data entry, management, and cleaning.

**Results:**

Productivity was higher in paper than digital methods in each round for each risk factor. However, the sample sizes across both methods provided a precision of 0.7 percentage points or smaller. The gap between digital and paper data collection productivity narrowed across rounds, with correlations improving from *r*=0.27-0.49 to 0.89-0.96. Reliability in risk factor proportions was between 0.61 and 0.99, improving between the two rounds for each risk factor. The results of the logistic regressions were also largely comparable between the two methods. Differences in regression results were largely attributable to small sample sizes in some variable levels or random error in variables where the prevalence of the outcome was similar among variable levels. Although data collectors were able to complete an observation using paper more quickly, the digital dataset was available approximately 9 days sooner. Although fixed costs were higher for digital data collection, variable costs were much lower, resulting in a 7.73% (US $3011/38,947) lower overall cost.

**Conclusions:**

Our study did not face trade-offs among time efficiency, cost efficiency, statistical reliability, and descriptive comparability when deciding between digital and paper, as digital data collection proved equivalent or superior on these domains in the context of our project. As trade-offs among cost, timeliness, and comparability—and the relative importance of each—could be unique to every data collection project, researchers should carefully consider the questionnaire complexity, target sample size, implementation plan, cost and logistical constraints, and geographical contexts when making the decision between digital and paper.

## Introduction

### Background

Road traffic injuries (RTIs) are a major global public health problem. With over 1.35 million deaths and 20-50 million nonfatal injuries estimated each year, RTIs impact all age groups and populations of all socioeconomic backgrounds [1]. However, low- and middle-income countries (LMICs), with rapid motorization in an unsafe road environment, bear a disproportionate share of deaths and disability [1]. In addition to safe vehicles, road infrastructure, and road safety management capacity, enhancing safe road user behavior plays an important part in preventing crashes and injuries [2]. The development and successful implementation of comprehensive programs to positively impact speeding, helmet, and seatbelt wearing require rigorously monitoring the prevalence of behavioral risk factors. One such example is the Bloomberg Initiative for Global Road Safety (BIGRS), a multisectoral program that unites a consortium of partners that work together to reduce the burden of RTIs in 10 cities in LMICs [3]. Roadside observational studies have played a fundamental role in designing evidence-informed strategies, including media campaigns, enforcement, and environmental modifications to enhance road safety.

Paper-based data collection has been the standard method for primary observational studies, but due to the possibility of human errors, storage costs, and time and labor required for double data entry, digital data collection methods are gaining popularity. Two concerns frequently influence the decision to switch from paper to digital methods of data collection. First, whether digital data collection is as productive and reliable as paper data collection in a dynamic roadside environment, and second, whether digital data collection is at least as efficient as paper data collection. Recent observational studies of road safety risk factors in three different countries, using both approaches have consistently demonstrated reliable results between the two methods [4]. In LMICs, with limited resources and relatively less expensive labor costs, paper data collection has been traditionally deemed as feasible and potentially more affordable. However, the efficiency of digital data collection vs traditional paper-based approaches has not been empirically assessed in these settings.

Efficiency is a rate measure against cost, time, or accuracy, and is used to assess whether the desired output can be produced in less time, using fewer resources, or with fewer errors. Efficiency is contextual, as cost and time efficiency is tied to the availability of infrastructure or other logistical requirements, as well as human and material resources. For a researcher deciding between using digital or paper data collection, provided there is a reasonable level of reliability and comparable productivity between the two methods, the decision may be based on factors related to efficiency.

### Objectives

We conducted an observational study in Mumbai, India, to understand the reliability, productivity, and efficiency of paper vs digital data collection based on three different road user behaviors: helmet use, seatbelt and child restraint use, and speeding. We also conducted a cost and time comparison to understand the relative efficiency of paper vs digital data collection across successive rounds, and to evaluate the potential trade-offs among different dimensions of efficiency (Table 1).

**Table 1:** Three areas of comparison between digital and paper data collection: productivity, reliability, and efficiency.

Dimensions of each area of comparison		Methods of measurement
**Productivity**		
	Volume	Number of observations per observation session
	Precision	Margin of error for estimation of proportions Akaike Information Criteria in regression analysis
**Reliability**		
	Statistical reliability	Proportion of risk factor by date, time, and location, as well as vehicle, occupant, and environmental characteristics
	Comparability of results	Adjusted odds ratios for vehicle, occupant, and environmental risk factors
**Efficiency**		
	Cost	Per survey Per dataset to achieve a certain level of precision Per labor-hour of time (may be differential by skill level and cost of labor)
	Time	Per survey Per complete dataset (preparation, data collection, data entry and verification, data management and cleaning)

## Methods

### Setting

This comparative study was conducted in Mumbai. Mumbai is India’s most populous city, as well as the country’s financial center and commercial capital [5]. Mumbai city has a population of approximately 12.5 million, with another 20.6 million people in the metropolitan area [6]. Mumbai has a high literacy rate, close to 90%, with 15.8 million internet subscribers [7,8]. Mumbai ranks sixth among major cities in India for number of road traffic collisions, with over 82% of all crashes resulting in at least one injury and almost 15% of collisions resulting in at least one fatality [9]. The registered vehicle fleet was 2,571,000 in 2015 [10].

### Data Collection

As part of the BIGRS project, the Johns Hopkins International Injury Research Unit partnered with the Indian Institute of Technology, Bombay to conduct semiannual observational studies to measure the prevalence of helmet use, seatbelt and child restraint use, and speeding at representative locations throughout the city. The details of the roadside data collection protocol are provided elsewhere [4]. To assess reliability between the two methods, paper and digital data collection were conducted simultaneously between January-March 2018, and again between July-September 2018. In each round, 75 of 150 observation sessions had simultaneous paper and digital data collection for each risk factor, which was used for our study analysis. Consistent procedures and definitions were maintained between methods, across observation sites, and across rounds to ensure comparability of results.

Observations on helmet and seatbelt use were conducted at intersections while vehicles were stationary to allow field teams to observe the use of safety equipment, whereas speeding observations were conducted at unobstructed stretches of road. Data were captured by an observer, who kept his or her attention on the flow of traffic and reported observations to a recorder, who wrote or entered into a tablet the road safety data. During the initial years of the project, the entire data collection was paper-based, while pilot testing of digital data collection in Mumbai started in 2018.

Each observation session began by capturing information about the site and observation session. These included (1) date, time of day, and location of each observation period; (2) names of the observer and recorder for that session; (3) the weather; (4) the volume of traffic over time; and (5) the presence of any law enforcement during the session. Paper-based data collection was handwritten into forms with a predesigned grid appropriate to each risk factor. Each row captured information on a single vehicle. For speeding observations, the columns captured road safety behavior for each vehicle, whereas for helmet and seatbelt use observations, occupant position, demographics, and road safety behavior were captured in a set of columns for each occupant in the vehicle.

The digital data collection tool was developed to mirror the paper-based tool, capturing all the same information, but in a series of screens through which the recorder would swipe to record information on each vehicle and vehicle occupant. For the BIGRS project, we used KoBoToolbox for digital data collection, an open-source mobile data collection platform [11]. The digitization process involved programming digital forms that were then downloaded onto the KoBoCollect mobile app. The content of both paper and digital questionnaires was the same; however, the digital form included mandatory fields, logic checks, and constrained text entry that prevented recorders from leaving fields blank or entering unreasonable or inconsistent responses. The team used Android tablets for data collection and uploaded the questionnaire forms to a secure cloud server at the end of each session.

Local data collectors were trained for two days on study protocols, paper data entry, the use of Android tablets and the KoBoCollect app, and how to upload data to the server. Supervisors and data managers were trained to monitor field site data collection and data aggregation on the server.

### Statistical Analysis

Helmet use was categorized as correctly, incorrectly, or not used. Correct helmet use was defined as using a strapped, standard helmet, whereas incorrect helmet use involved either wearing a *cap* or *tropical* helmet, or using an unstrapped helmet. Neither digital or paper data collectors would be able to distinguish an imitation or substandard helmet from a genuine standard helmet, as that would require close inspection for a standards label. Although this may slightly inflate the proportion of the sample assessed to be wearing a helmet correctly, it would not do so differentially by data collection method, and so should not affect measures of comparison between the two methods. Correct seatbelt use was defined as the use of age-appropriate restraints: seatbelts for adults and child or booster seats for children under 12 years old. Incorrect and no restraint use were grouped due to the low occurrence of incorrect restraint use. Speeding was defined as any excess of the speed limit or was grouped categorically as not speeding, speeding up to 10 kilometers per hour (kph), and speeding over 10 kph above the posted limit.

For helmet use observations, the age variable captured whether the occupant was observed to be over or under the age of 18, whereas for seatbelt use observations, a greater level of detail was captured to assess whether children under 12 years were using age-appropriate child restraints. During the analysis, we did not distinguish children under 12 by gender, as observers were not able to reliably determine a child’s gender through observation. Helmet use observations categorize motorcycle riders as drivers or passengers, whereas seatbelt use observations distinguished passengers further as sitting in the front or rear seats. Sex was captured as either male or female for both helmet and seatbelt use observations. Speeding observations did not capture any occupant characteristics, as data collectors cannot accurately observe inside vehicles in motion. For speeding and seatbelt use observations, the vehicle type and ownership were captured; vehicle types were either a sedan, sport utility vehicle, pickup or light truck, minivan, heavy truck, bus, or, for speeding observations only, motorcycle. Vehicles ownership was classified as private, government, commercial, taxi, or tourist vehicles, based on markings on the outside of the vehicle, particular to the context. Vehicle type and ownership were not included in helmet use observations, as all vehicles were uniformly private motorcycles.

We matched simultaneous digital and paper observation periods by date, time, and location. We compared the productivity of the two methods by calculating the correlation in the number of observations made on vehicles (for speeding) or vehicle occupants (for helmet and seatbelt use) per 90-min observation session. The measure for reliability was the correlation in the prevalence of each risk factor among subgroups defined by vehicle occupant, vehicle, and environmental characteristics within matching sessions. Both reliability and productivity were assessed using Pearson correlations. Although Spearman rank correlations are more conventionally used to compare proportions, we were interested in how closely the proportions exactly matched each other, rather than whether the rank ordering of proportions was similar between digital and paper data collection methods. Pearson correlations are also appropriate for proportions when the proportions are not close to 0 or 1. We also performed a precision analysis, evaluating the margin of error available with current data and the sample size that would be required to estimate a proportion within 1 and 2 percentage points.

We also wished to understand whether the method of data collection would influence the results of descriptive or inferential analysis. To achieve this, we aggregated together all observation sessions by data collection method and round of observation (January-March 2018 and July-September 2018), creating four datasets. We conducted descriptive analysis separately within each dataset and compared the prevalence of various risk factors, overall and across subgroups. We also conducted multivariable logistic regression separately using each of the four datasets and compared the adjusted odds ratios.

Covariates included in the regressions included the occupant sex, age, and position in or on the vehicle, vehicle type, and ownership. Also included were the time of day, the location, and whether the date was a weekend or weekday. Finally, environmental factors included whether there was visible police presence, camera enforcement, or, for speeding observations, speed deterrents such as speed bumps, stop signs, or crosswalks. For analysis related to speeding, occupant characteristics were not included. As the location of the observation being naturally collinear with the presence of environmental speed deterrents, we included speed deterrents in our models and omitted locations, as the effect of environmental deterrents on speeding is of interest to road safety researchers.

### Cost Estimates

Personnel costs included field data collectors (observers, recorders, and field supervisors) and data entry operators, and logistical costs included staff training and transportation to observation sites. Supplies and equipment included running costs for office supplies, space for paper data entry, data plans for tablets, with one-time costs for computers for data entry, tablets and power banks, and app development. We assumed that tablets and power banks would need to be replaced every 3 years. Digital data collection running costs also included annual server costs and cloud data backup. Costs were calculated using the 2018 midyear exchange rate between Indian rupees (INR) and US dollars. Costs were calculated for only the 75 observation sessions that were simultaneously collected using both paper and digital methods.

### Time Estimates

The time to complete an observation on a vehicle (for speeding observations) or on the vehicle occupants (for helmet use and seatbelt use observations) was assessed by dividing the 90 minutes of each observation session by the volume of observations in that session, and then multiplying by 60 seconds for a minute to obtain the number of seconds needed to complete an observation. Finally, we took the average number of seconds to complete an observation for each risk factor in each round of data collection.

The time to obtain a complete dataset was measured as the number of days it took until the dataset was entered, cleaned, and ready for analysis, which included all work performed by the local partners onsite, including training, field work itself, data entry and verification of paper data, and both local and offsite data cleaning and management. As only half of the paper sessions were simultaneously collected digitally, time estimates for paper data entry and verification were appropriately divided in half.

## Results

### Productivity Comparison

**Figure 1:**
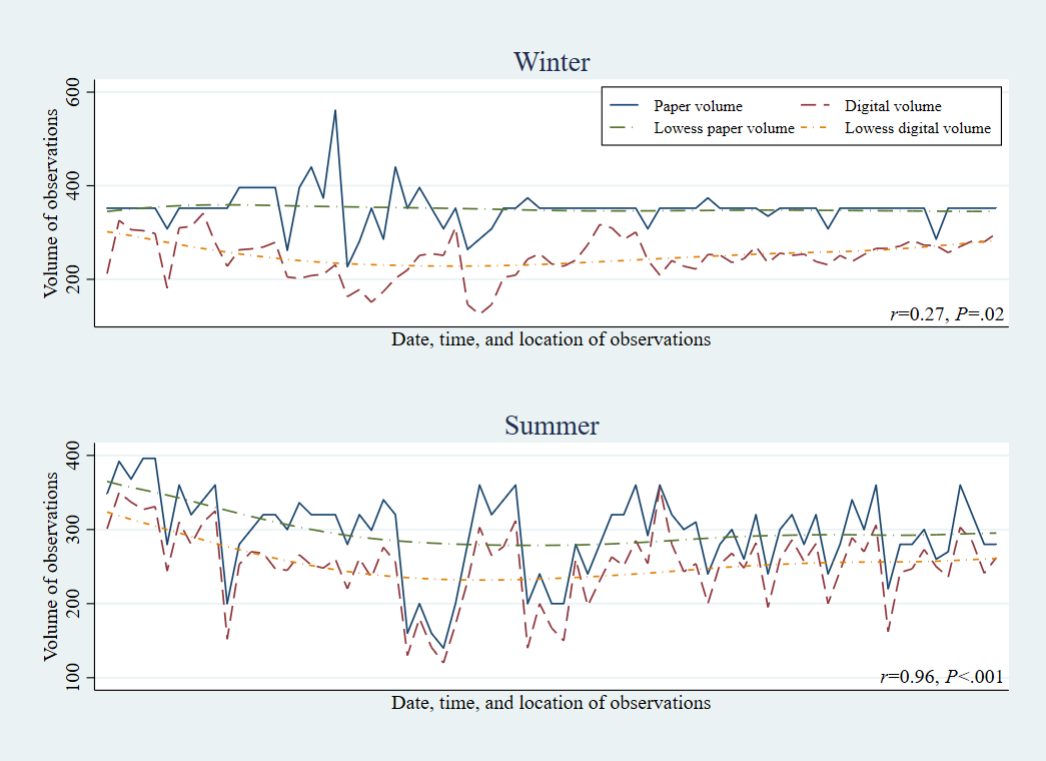
Correct helmet use: correlation between digital and paper volumes of observations by round.

**Figure 2:**
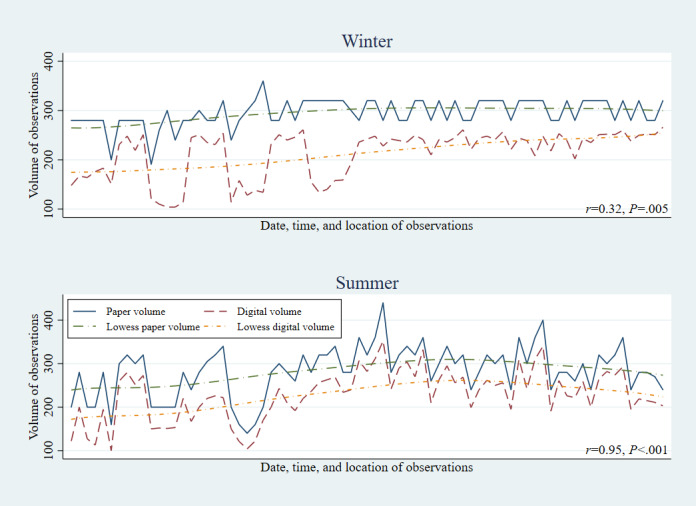
Seatbelt use: correlation between digital and paper volumes of observations by round.

**Figure 3:**
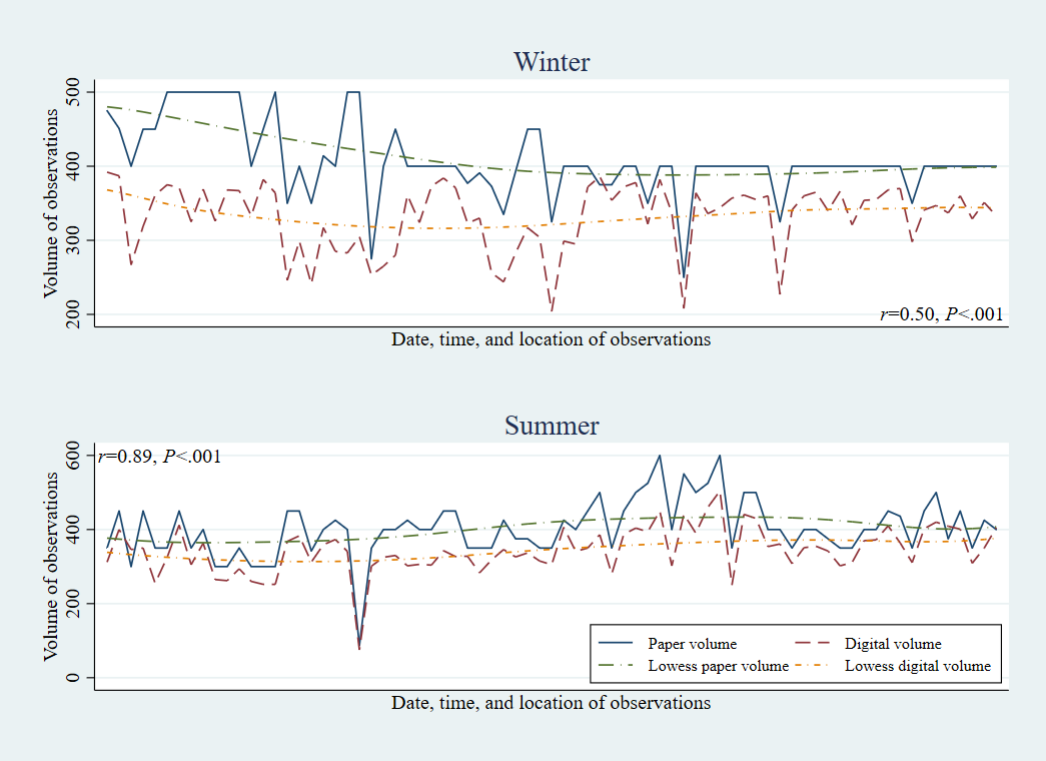
Speeding: correlation between digital and paper volumes of observations by round.

Across each of the three risk factors, paper methods showed higher productivity than digital methods, in both the winter (January-March) and summer (July-September) data collection rounds (Figures 1-3). However, the gap between digital and paper data collection volumes narrowed between the winter and summer rounds, with the correlation in productivity by observation session increasing from r=0.27 to 0.96 among helmet use, 0.32 to 0.95 among seatbelt use, and 0.49 to 0.89 among speeding observations.

The results of our precision analysis showed that the existing sample sizes in digital and paper provided precision to within 0.7 percentage points or smaller (1.4% margin of error). Conversely, the difference between paper and digital levels of precision was less than 0.2 percentage points, regardless of risk factor or round of data collection (Table 2). When comparing results from the regression analysis, digital data collection had a lower Akaike Information Criteria (AIC) in all cases except in the summer speeding data collection. This was despite digital data collection methods having a smaller sample size and the same number of variables in the models (Multimedia Appendix 1).

**Table 2:** Level of precision: current and needed sample sizes.

Risk factor and round of data collection		Paper sample size, n	Precision with existing paper sample, proportionate terms	Digital sample size, n	Precision with existing digital sample, proportionate terms	Required sample size for estimation within 1 percentage point (0.01), n	Required sample size for estimation within 0.5 percentage points (0.005), n
**Helmet use**							
	Winter	34,309	0.006	24,283	0.007	9595	38,377
	Summer	29,286	0.006	24,778	0.007	9585	38,338
**Seatbelt Use**							
	Winter	36,573	0.006	25,452	0.007	9603	38,411
	Summer	35,434	0.006	27,423	0.006	9604	38,414
**Speeding data**							
	Winter	30,634	0.004	24,799	0.006	7827	31,307
	Summer	30,190	0.004	25,783	0.006	8459	33,835

### Statistical Reliability

Reliability in the prevalence of behavioral risk factors matched by date, time, location, and by characteristics of vehicles and vehicle occupants showed moderate to high levels of correlation in both rounds of data collection. There were some improvements in correlations between the winter and summer rounds of data collection among all risk factors, particularly among speeding observations and in the prevalence of incorrect helmet use (Table 3).

**Table 3:** Reliability in behavioral risk factor prevalence: Pearson correlation and *P* value.

Risk factor		Winter		Summer	
		Correlation value (*r)*	*P* value	Correlation value (*r)*	*P* value
**Helmet use^a^**					
	Any helmet use	0.98	<.001	0.99	<.001
	Correct helmet use	0.82	<.001	0.86	<.001
	Incorrect helmet use	0.79	<.001	0.92	<.001
**Seatbelt use^a^**					
	Correct seatbelt use	0.82	<.001	0.82	<.001
**Speeding^b^**					
	No speeding	0.73	<.001	0.80	<.001
	≤10 kph^c^ over speed limit	0.61	<.001	0.63	<.001
	>10 kph over speed limit	0.72	<.001	0.73	<.001

^a^Matched by date, time, location, age, sex, and position.

^b^Matched by date, time, location, vehicle type, and ownership.

^c^kph: kilometers per hour.

### Overall Comparison: Risk Factor Proportions

The descriptive analyses showed similar results between methods for most subgroups defined by vehicle and occupant characteristics. For helmet observations, the digital and paper datasets showed correct helmet use proportions within 2 percentage points of each other, across subgroups by age, sex, and role or position. Among seatbelt observations, digital and paper seatbelt use prevalence by vehicle type and occupant position were within 5 percentage points of each other, except for those subgroups with fewer than 200 observations. For comparisons of speeding by vehicle ownership, digital and paper percentages within each category of speeding were also within 5 percentage points of each other. Across all risk factors, the comparability decreased as the subgroup sample size decreased (Multimedia Appendices 2-4).

### Overall Comparison: Regression Results

**Figure 4:**
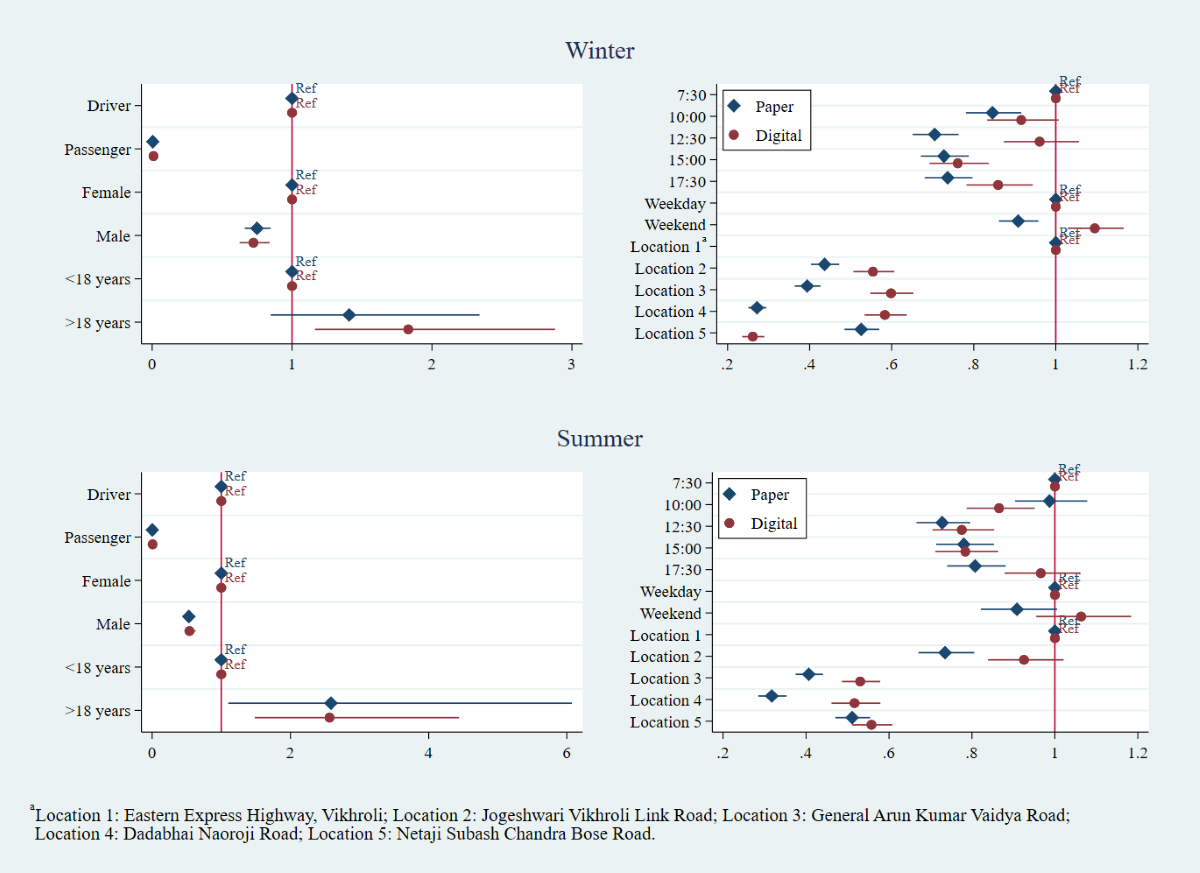
Correct helmet use: adjusted odds ratios by data collection method and round.

The multivariate logistic regressions for correct helmet use, correct seatbelt use, and any speeding showed more variability. Estimates for adjusted odds ratios were largely similar for the associations between occupant characteristics (age, sex, and position) and correct helmet use, regardless of method or round of data collection, adjusting for the day, time, and location (Figure 4). Passengers, compared with drivers, had consistently lower odds of using a helmet correctly, as did males, compared with females, and occupants under 18 years of age, compared with those over 18 years of age. However, the age estimate in the winter 2018 paper dataset was not significant, leading to a different inference from the digital dataset about the impact of age on helmet use between digital and paper. By contrast, the adjusted odds ratios for day of week, time, and location of data collection did not have as many overlapping CIs in either round of helmet use data collection.

**Figure 5:**
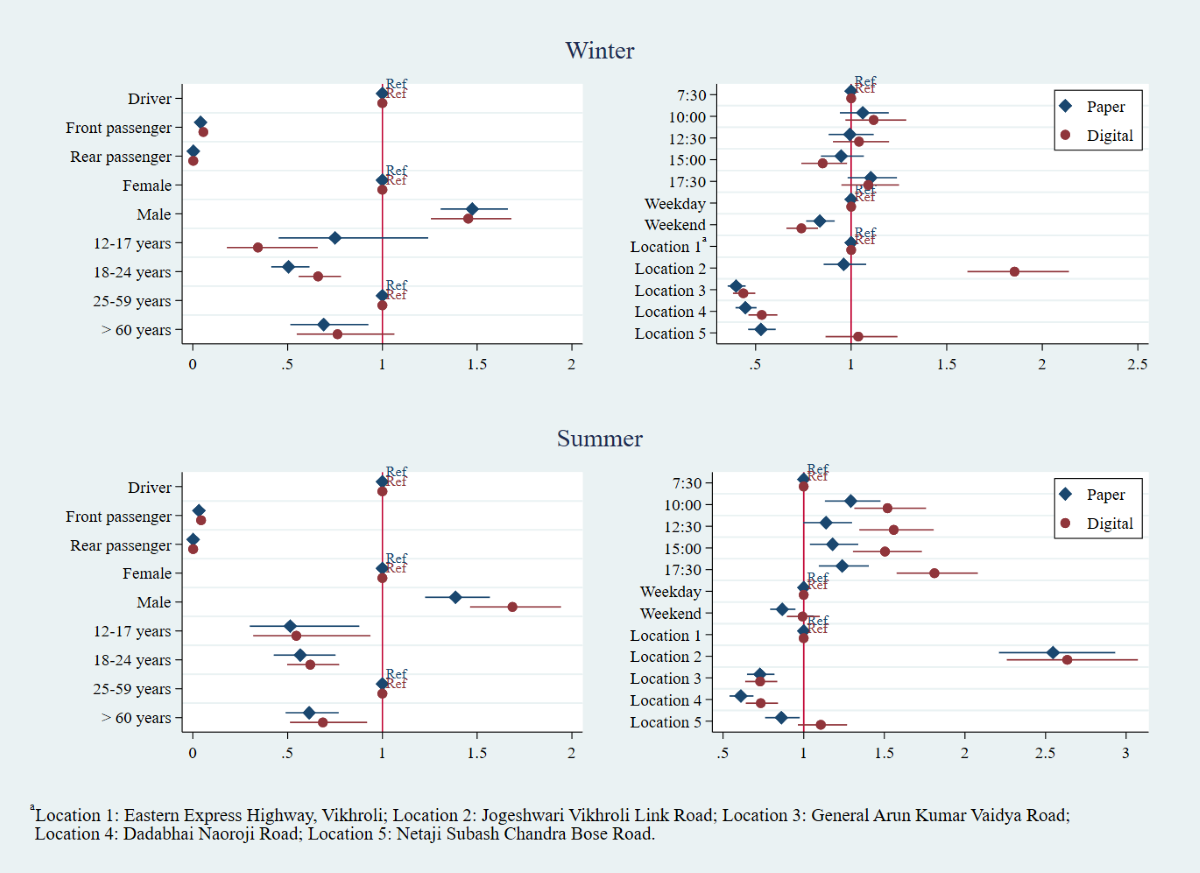
Seatbelt use: adjusted odds ratios by data collection method and round.

As with helmet use data collection, the relationships between occupant characteristics and seatbelt use were comparable between digital and paper datasets, while adjusted odds ratios for day of week, time, and location showed more variability (Figure 5). We regressed sex, age, occupant position, day, time, location, vehicle type, and vehicle ownership onto correct seatbelt use. Seatbelt use was higher among males as compared with females, with front and rear passengers showing very low odds of seatbelt use, as compared with drivers, in both paper and digital datasets. There were similar estimates with overlapping CIs for all occupant age groups, although inferences differed between data collection methods for some age groups (12-17 years and over 60 years) in the winter round of data collection, as the paper data collection CI overlapped with the null value.

**Figure 6:**
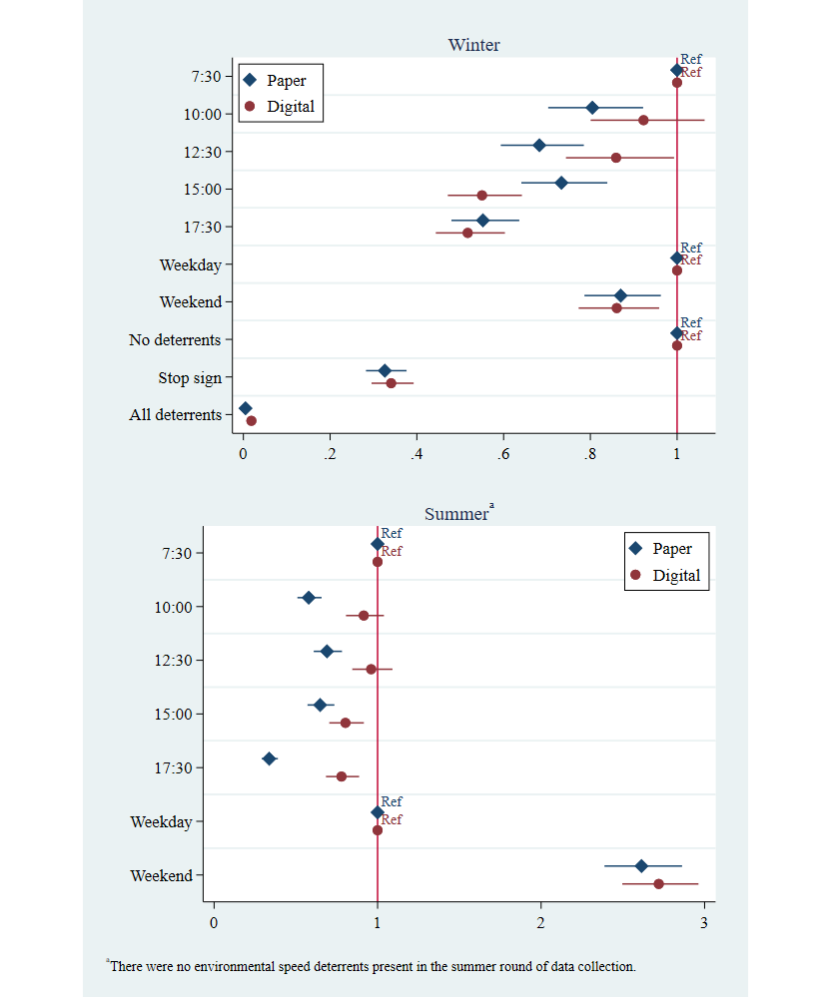
Speeding: adjusted odds ratios by data collection method and round.

Similar to the other two risk factors, in the speeding regressions, the adjusted odds ratios for time showed some variability, controlling for vehicle type, vehicle ownership, and environmental speed deterrents (Figure 6). The effect of speed deterrents, which were only present during the winter round of data collection, showed a similar impact on reducing speeding regardless of data collection method; both the magnitude of the adjusted odds ratios and inferences were similar. Please see Multimedia Appendices 5-7 for further details on regression results for all risk factors.

### Efficiency: Cost Comparison

A comparison of the initial set up and running costs for paper and digital data collection for this study is presented in Table 4. Field data collection costs for staffing and transportation were identical between methods. The field data collection for a single round was covered in 2880 labor hours for each method. Fixed costs or one-time costs, for digital and paper data collection, differed. Four android tablets with sim cards, cases, and power banks were used for digital data collection. For the paper data collection, two desktop computers with hard drives were purchased in the first round, and a laboratory space for paper data entry and storage was rented.

Variable costs or running costs for materials and supplies in paper data collection included paper printing, pens, and clipboards. For digital data collection, variable costs included monthly cellular data plans to ensure continuous connectivity and annual subscription of server space and backup storage. The variable costs for labor were also different between digital and paper data collection methods. Data management for paper data included data entry, cleaning, and verification. Data entry required 450 labor hours divided over 5 persons, with data cleaning and verification taking an additional 180 labor hours. This could take an additional 26.25 days of work per person, an estimated 105 labor days for 4 data entry operators working 6 hours per day. Digital data management included initial app development, data cleaning, and daily server monitoring, taking approximately 12 labor hours per round.

During the first round, digital data collection cost 1.54% (US $310/20,071) more than paper data collection, driven by the fixed costs of software development and server setup. However, over both rounds in year 1, digital data collection cost 7.73% (US $3011/38,947) less than paper data collection, as with further data collection the higher variable costs in paper exceed the higher fixed costs with digital data collection. Given that the project would potentially last for multiple years, projected costs for data collection over 4 years is presented in Table 5. Over 4 years with two rounds of data collection per year, digital data collection would result in 10.03% (US $15,268/152,203) cost savings, primarily by cutting data entry and verification costs.

**Table 4:** Cost comparison between paper and digital data collection across rounds of data collection in US dollars.

Cost description			Paper			Digital		
			Units	Winter, US $	Summer, US $	Units	Winter, US $	Summer, US $
**Fixed costs**								
	**Hardware**							
		Computers for data entry, number	2	1195	N/A^a^	N/A	N/A	N/A
		Tablets and accessories, number	N/A	N/A	N/A	4	1972	N/A
	**Others**							
		Laboratory space for data entry and storage, total cost	1	299	299	N/A	N/A	N/A
		App development, labor hours	N/A	N/A	N/A	20	955	N/A
		Server and back up, annual total cost	N/A	N/A	N/A	1	1899	N/A
	**Training**							
		Total training costs, days	2	299	299	2	299	299
**Variable cost**								
	**Data collection personnel**							
		Observation, labor hours	1260	5647	5647	1260	5647	5647
		Recording, labor hours	1260	5647	5647	1260	5647	5647
		Supervision, labor hours	360	2690	2690	180	1345	1345
	**Data Management**							
		Data entry, labor hours	450	1008	1008	N/A	N/A	N/A
		Data cleaning and verification, labor hours	180	1076	1076	12	364	364
	**Others**							
		Supplies (pens, paper, etc.), total cost	1	195	195	N/A	N/A	N/A
		Tablet data plans, days	N/A	N/A	N/A	360	239	239
		Transportation, trips	180	2017	2017	180	2017	2017

^a^N/A: not applicable. This type of cost was not incurred.

**Table 5:** Realized and projected cost savings by switching to digital data collection in US dollars.

Cost description over years		Winter			Summer			Yearly savings from DDC^a^
		Paper	Digital	Paper		Digital		
**Year 1 (realized)**								
	Fixed costs, US $	1793	5125^b,c^	598		299	N/A^d^	
	Variable costs, US $	18,278	15,256	18,278		15,256	N/A	
	Total costs, US $	20,071	20,381	18,876		15,555	N/A	
	Cost savings from DDC^e^ (US $), n (%)	N/A	–310 (–1.54)	N/A		3321 (17.59)	3011 (7.73)	
**Year 2 (projected)**								
	Fixed costs, US $	598	2198^b^	598		299	N/A	
	Variable costs, US $	18,278	15,256	18,278		15,256	N/A	
	Total costs, US $	18,876	17,454	18,876		15,555	N/A	
	Cost savings from DDC^e^ (US $), n (%)	N/A	1422 (7.53)	N/A		3321 (17.59)	4743 (12.56)	
**Year 3 (projected)**								
	Fixed costs, US $	598	4170^b,f^	598		299	N/A	
	Variable costs, US $	18,278	15,256	18,278		15,256	N/A	
	Total costs, US $	18,876	19,426	18,876		15,555	N/A	
	Cost savings from DDC^e^ (US $), n (%)	N/A	–550 (–2.91)	N/A		3321 (17.59)	2771 (7.34)	
**Year 4 (projected)**								
	Fixed costs, US $	598	2198^b^	598		299	N/A	
	Variable costs, US $	18,278	15,256	18,278		15,256	N/A	
	Total costs, US $	18,876	17,454	18,876		15,555	N/A	
	Cost savings from DDC^e^ (US $), n (%)	N/A	1422 (7.53)	N/A		3321 (17.59)	4743 (12.56)	
Total cost savings (projected; US $), n (%)		N/A	N/A	N/A		N/A	15,268 (10.03)	

^a^DDC: digital data collection.

^b^Includes yearly subscription of the cloud server.

^c^Includes initial app development.

^d^N/A: not applicable.

^e^This is difference between paper and digital costs as a percentage of the paper cost ([paper–digital]/paper).

^f^Assumes replacement of tablets and accessories after 2 years.

### Efficiency: Time Comparison

As mentioned earlier, productivity was higher in paper collection as compared with digital data collection, with improvement in the correlation of volumes over time. With observation periods lasting 90 min in each data collection method, this higher productivity in paper data collection is due to the fact that it took field teams between 2-6 seconds longer to collect data digitally than to collect data using paper forms, on average (Table 6). Such small differences added up over the several hundred observations made per observation period and over 75 observation periods per round. Interestingly, the improvement in the correlation of productivity between the winter and summer rounds was due not only to an increase in speed in digital data collection but a slight drop in speed in paper data collection. Between the winter and summer data collection rounds, the time to complete an observation decreased or remained the same in digital but increased slightly in paper.

Although each observation took less time in paper data collection, it took less time to obtain a complete dataset with digital data collection. The time for training and field data collection were the same between digital and paper data collection methods, with 2 days for training and 15 days for field data collection. Data entry and verification in paper data took an additional 630 hours, or 26.25 days for 4 data entry operators to complete, which was done concurrently with ongoing fieldwork. Under the best conditions, this would delay receipt of data collection by a minimum of 11 days after fieldwork was complete. By contrast, digital data collection initially required 20 hours before fieldwork to develop the app and set up the server, and an average of 12 hours of data cleaning following receipt of the data before it was ready for analysis. Switching from paper to digital data collection reduced the time to receive a clean dataset after fieldwork was completed from 630 hours to 12 hours, or from 11 days to 2 days.

**Table 6:** Time in seconds to complete an observation by data collection method and round.

Risk factor	Winter, time (seconds)		Summer, time (seconds)	
	Paper	Digital	Paper	Digital
Helmet use^a^	12.0	17.6	14.6	17.4
Seatbelt use^a^	11.3	17.5	12.4	16.4
Speeding^b^	13.4	16.7	14.3	16.7

^a^Observation on a vehicle occupant.

^b^Observation on a vehicle.

## Discussion

### Productivity

In our study, productivity was higher in paper data collection as compared with digital data collection regardless of the round of data collection or risk factor. The majority of studies comparing productivity between digital and paper data collection methods found equivalent or less time to complete a digital survey or observation, as compared with paper data collection [12-16]. Paper data collection may have taken less time in our study for several reasons. First, the data collectors had greater familiarity and experience with paper data collection, having previously completed five rounds of paper-based data collection over 3 years. Second, the act of swiping through multiple screens during digital data collection may take more time than filling in a single row in a predesigned table format on paper. Third, the digital tools used logic checks and constraints to prevent errors, which may delay recording the response. Although digital productivity did improve over time, it is not possible to project whether it would ever eventually catch up to the productivity seen in paper data collection, due to the formatting of the digital module and the logic checks imposed. Researchers and project managers may wish to spend additional time on classroom and field training with digital data collection tools, to give field staff the extra practice required to close this productivity gap as much as possible.

Despite the higher productivity in paper data collection, both digital and paper data collection methods were able to obtain a large enough sample size to estimate a proportion within ± 1 percentage point, which we judged to be a reasonable margin of error. Moreover, this higher productivity in paper did not translate into higher quality statistical models, measured using AIC.

### Reliability

Similar to other direct comparisons of digital and paper data collection, we found high reliability between the two methods [15,17-27]. This high reliability was seen across risk factors, and improved between rounds of data collection, perhaps indicating improvements in data collector facility with the digital modules over time.

### Overall Comparison: Risk Factor Proportions and Regression Results

The descriptive analyses showed comparable results of behavioral risk factor prevalence between subgroups defined by occupant, vehicle, or environmental characteristics, but with comparability decreasing with sample size. Other studies that conducted descriptive analyses also found similar distributions between data collection methods [12,15-28].

However, the multivariable regression analyses showed that the digital and paper datasets produced comparable results for some variables but not for others. Occupant characteristics—age, sex, and position—showed similar adjusted odds ratios, meaning that a researcher using digital data collection would have come to the same conclusions as a researcher using paper-based data collection when trying to understand the occupant-related factors associated with the use of protective equipment. However, there was less comparability between digital and paper methods in odds ratios for vehicle-related factors, or for day, time, and location, across risk factors.

Owing to smaller sample sizes in some subgroups, some age categories, vehicle types, and vehicle ownership types showed different prevalence estimates between paper and digital methods. Differences in regression results by day, time, and location, between digital and paper methods seem to be due to the nature of observational sampling and the differences in productivity between digital and paper methods. With faster data collection in paper, digital and paper data collectors do not make observations on the *same* target vehicles and their occupants. Such random differences are less pronounced for occupant characteristics (age, sex, or position), or when the prevalence of the risk factor are significantly modified by sex and occupant roles (drivers vs passengers). Differences in risk factor prevalence are less pronounced among the different locations and times of day, and therefore, random differences in target vehicle selection may have a larger effect on prevalence and odds ratio estimates. These results cannot be explained by surveyor effects, as the pool of data collectors were randomly assigned to data collection teams, and teams were randomly switched between methods and among locations, times of day, and days of the week.

### Efficiency: Cost Comparison

Digital data collection had higher fixed costs and lower variable costs compared with paper data collection. In the initial round of data collection, digital data collection was slightly more expensive, driven by the costs of purchasing tablets, but became less expensive with additional rounds of data collection. Digital data collection is significantly more cost efficient than paper when costs are annualized over several years with ongoing data collection. Digital data collection will have the greatest cost efficiency when used by projects with larger sample sizes, or multiple and extended rounds of data collections [29]. As labor, training, and transportation cost would be the same between methods, regardless of annual salary increase or inflation rate, the cost savings by digital data collection are driven primarily by savings in data management, particularly data entry. Other studies have similarly found higher fixed costs for digital data collection and lower variable costs, providing increased cost savings for larger sample sizes [13,14,16,30-33].

Cost differences between digital and paper will vary by project and context. The relative cost of labor, hardware, and electricity may differ by geographic location. Additionally, the cost of data collection platforms differs by company and by project. For example, for this project, the server space was purchased to accommodate data for all 10 cities, and hence the actual space required for Mumbai data was smaller and could have cost significantly less. Contractual agreements and price negotiations for server space could be influenced by a number of factors such as the size of the company, infrastructure and service details, promotions, packages, size and configuration of the server space, program, data security and back up arrangements, length of time, region, and location. The cost figures provided are particular to this project, and we encourage researchers for whom cost differential is a major consideration to draw up similar comparative budgets for digital and paper data collection methods for a given project. On the basis of our experience and the existing literature, for each study, there will be a sample size threshold above which digital data collection will provide cost savings, and below which paper data collection will be the more cost-effective option.

Our study was not able to directly measure a third dimension of efficiency: accuracy, or the reduction in errors achieved per resource unit expended. However, we note that the error protection built into the digital app through logic checks and constraints had two advantages. It decreased the cost of digital data collection as compared with paper, as evident by significantly fewer hours spent on verification and cleaning of data. It also decreased the size of the AIC seen in digital data collection compared with paper, even with lower sample size in digital methods. In other words, digital data collection provided our project with more precision at a lower cost.

### Efficiency: Time Comparison

One of the biggest benefits of digital data collection is the real-time nature of data collection and availability: once data collection starts, the digital data are immediately available for data cleaning and analysis, while researchers using paper data collection must wait for data entry and reconciliation to be complete. It is important to note that for complex questionnaires, more time may be needed at startup for digital data collection as compared with paper-based data collection [24,34]. However, the efficiency for subsequent rounds of data collection would be significantly increased. The majority of studies comparing digital and paper data collection methods found that digital data collection reduced the time to obtain a complete dataset, as they avoided double data entry and reconciliation [14,16,33,35].

### Conclusions

Our study did not face trade-offs among time efficiency, cost efficiency, statistical reliability, and descriptive comparability when deciding between digital and paper methods, as digital data collection proved equivalent or superior on these domains in the context of our project. Digital data collection provides estimates which are precise and reliable with paper data collection, with overall comparable results. Digital data collection may take longer per survey but takes less calendar time to obtain a completed dataset. The setting, context, length of the survey, and desired sample size play a role in determining the extent of time and cost efficiency. However, we may have encountered a method effect due to sampling methods for observational studies and random error.

Researchers considering using digital data collection may benefit from developing comparative budgets for each of the two methods, and pilot testing each method to understand relative productivity. When working with data collectors who are unfamiliar with handheld devices or with the chosen digital data collection app, additional classroom and field training on the digital data collection tools would improve productivity. Although this may slightly narrow the time efficiency advantage that digital data collection has over paper and increase the cost of training for digital methods as compared with paper, this additional effort would be worthwhile especially in studies relying on higher productivity. As trade-offs among cost, timeliness, and comparability⸺and the relative importance of each⸺could be unique to every data collection project, researchers should carefully consider the questionnaire complexity, target sample size, implementation plan, cost and logistical constraints, and geographical contexts when making the decision between digital and paper [36].

## Appendix

Multimedia Appendix 1 Akaike Information Criterion for logistic regressions, by risk factor, round, and method of data collection.

Multimedia Appendix 2 Whole sample proportions of correct, incorrect and no helmet use by occupant role, age, and sex.

Multimedia Appendix 3 Whole sample proportions of restraint use by occupant role and vehicle type.

Multimedia Appendix 4 Whole sample proportion of vehicles speeding by vehicle ownership.

Multimedia Appendix 5 Correct helmet use: adjusted odds ratios and 95% CIs by round and method of data collection.

Multimedia Appendix 6 Seatbelt use: adjusted odds ratios and 95% CIs by round and method of data collection.

Multimedia Appendix 7 Speeding: adjusted odds ratios and 95% CIs by round and method of data collection.

## Abbreviations

AIC: Akaike Information Criteria
BIGRS: Bloomberg Initiative for Global Road Safety
kph: kilometers per hour
LMICs: low- and middle-income countries
RTI: road traffic injury
