# Supplementary material for: Paper Versus Digital Data Collection Methods for Road Safety Observations: Comparative Efficiency Analysis of Cost, Timeliness, Reliability, and Results
Source: J Med Internet Res. 2020 May 22;22(5):e17129. doi: 10.2196/17129 (PMC7275261; doi:10.2196/17129)
Supplement: Multimedia Appendix 1 [file jmir_v22i5e17129_app1.docx]

Multimedia Appendix 1. Akaike Information Criterion (AIC) for logistic regressions, by risk factor, round, and method of data collection.

|  | Winter | Winter | Spring | Spring |
| --- | --- | --- | --- | --- |
|  | Paper | Digital | Paper | Digital |
|  |  |  |  |  |
| **Helmet use** |  |  |  |  |
| Observations used | 34,308 | 24,253 | 29,286 | 24,749 |
| Degrees of Freedom | 13 | 13 | 13 | 13 |
| AIC | 35738.27 | 25554.75 | 29602.11 | 25883.60 |
|  |  |  |  |  |
| **Seatbelt Use** |  |  |  |  |
| Observations used | 36,384 | 25,307 | 35,170 | 27,193 |
| Degrees of Freedom | 25 | 25 | 26 | 26 |
| AIC | 20036.40 | 14188.79 | 18108.09 | 14584.07 |
|  |  |  |  |  |
| **Speeding** |  |  |  |  |
| Observations used | 28,964 | 23,965 | 28,107 | 24,392 |
| Degrees of Freedom | 18 | 18 | 16 | 16 |
| AIC | 13449.29 | 11891.60 | 15865.29 | 16734.40 |
