# Supplementary figures and images for: Paper Versus Digital Data Collection Methods for Road Safety Observations: Comparative Efficiency Analysis of Cost, Timeliness, Reliability, and Results
Source: J Med Internet Res. 2020 May 22;22(5):e17129. doi: 10.2196/17129 (PMC7275261; doi:10.2196/17129)

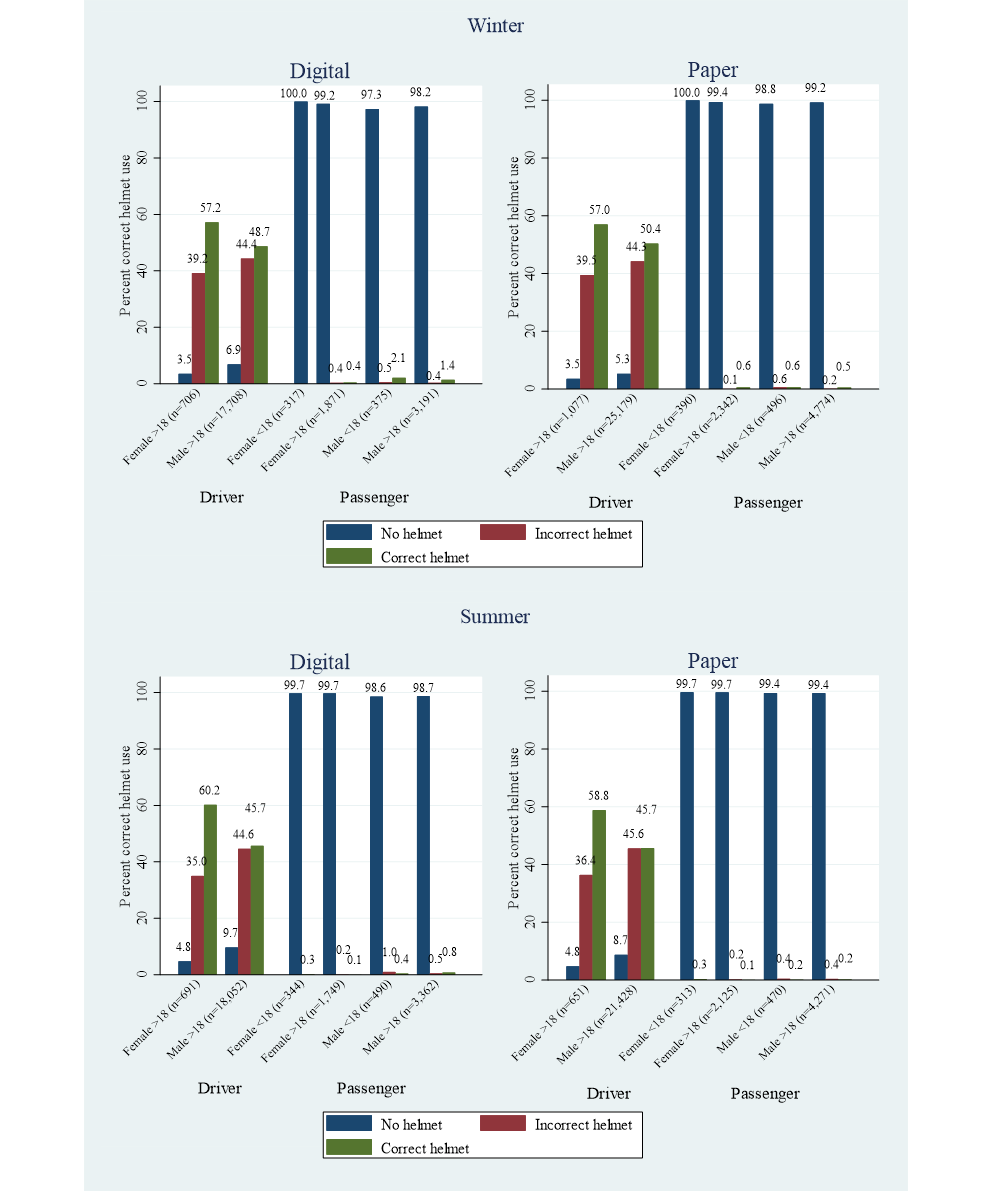

Supplement: Multimedia Appendix 2 [file jmir_v22i5e17129_app2.png]

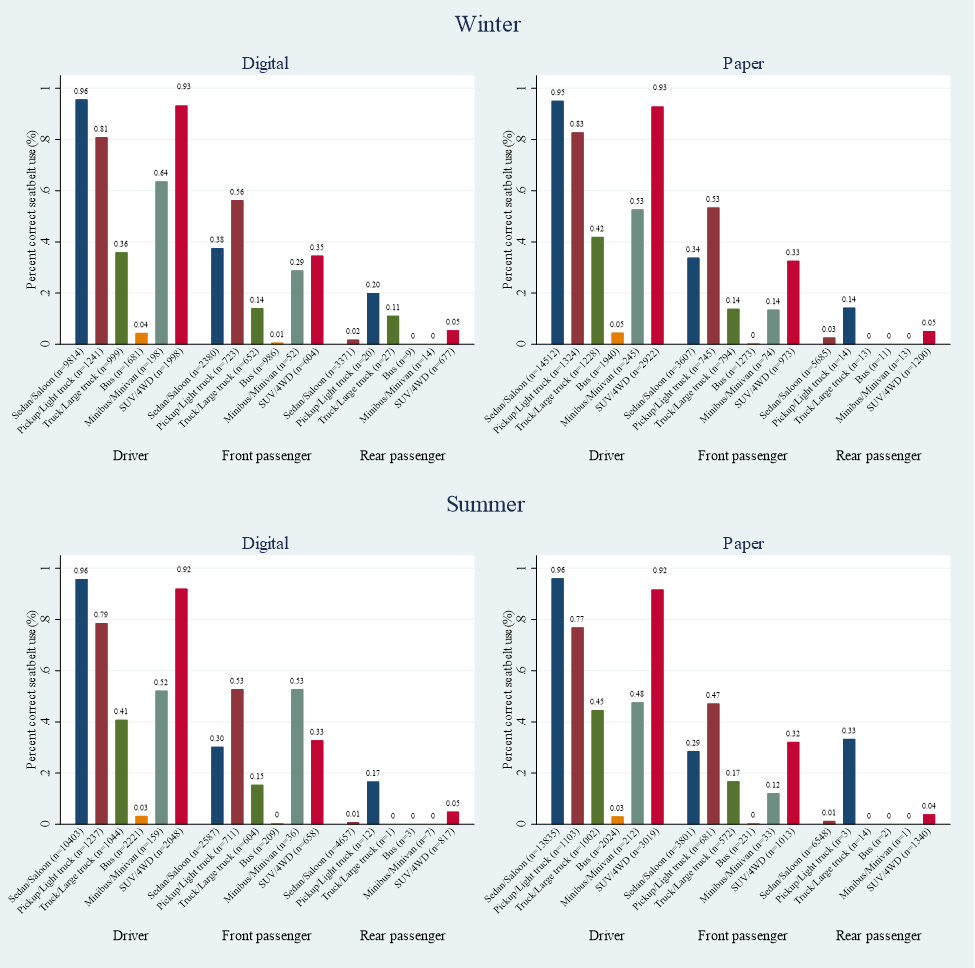

Supplement: Multimedia Appendix 3 [file jmir_v22i5e17129_app3.png]

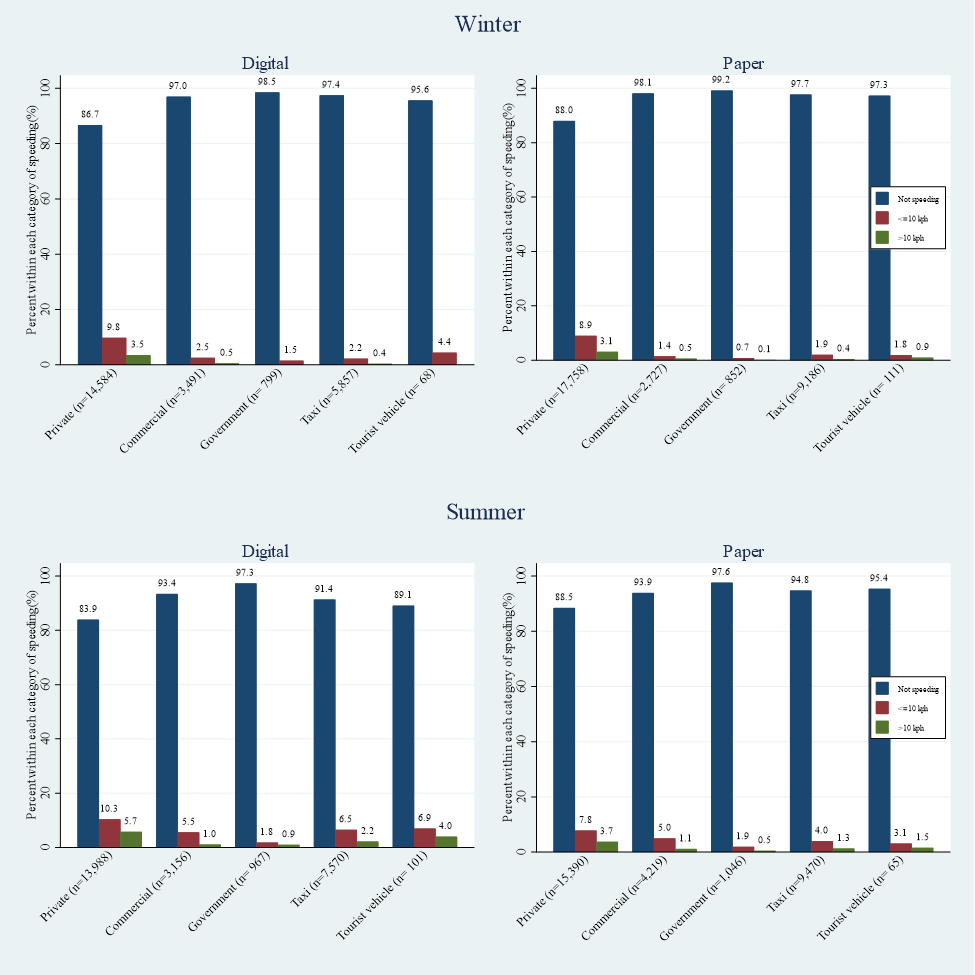

Supplement: Multimedia Appendix 4 [file jmir_v22i5e17129_app4.png]
