# Supplementary material for: Paper Versus Digital Data Collection Methods for Road Safety Observations: Comparative Efficiency Analysis of Cost, Timeliness, Reliability, and Results
Source: J Med Internet Res. 2020 May 22;22(5):e17129. doi: 10.2196/17129 (PMC7275261; doi:10.2196/17129)
Supplement: Multimedia Appendix 5 [file jmir_v22i5e17129_app5.docx]

Multimedia Appendix 5. Correct helmet use: adjusted odds ratios (aOR) and 95% confidence intervals (CI) by round and method of data collection.

| **Variable** | Winter | Winter | Summer | Summer |
| --- | --- | --- | --- | --- |
|  | Paper | Digital | Paper | Digital |
|  | aOR  (95% CI) | aOR  (95% CI) | aOR  (95% CI) | aOR  (95% CI) |
|  |  |  |  |  |
| **Occupant role** |  |  |  |  |
| Drivers | 1  (Ref) | 1  (Ref) | 1  (Ref) | 1  (Ref) |
| Passengers | 0.004  (0.003, 0.006) | 0.009  (0.007, 0.012) | 0.002  (0.001, 0.003) | 0.005  (0.004, 0.008) |
|  |  |  |  |  |
| **Occupant sex** |  |  |  |  |
| Female | 1  (Ref) | 1  (Ref) | 1  (Ref) | 1  (Ref) |
| Male | 0.75  (0.66, 0.85) | 0.72  (0.63, 0.84) | 0.53  (0.45, 0.62) | 0.54  (0.46, 0.63) |
|  |  |  |  |  |
| **Occupant age** |  |  |  |  |
| Less than 18 years | 1  (Ref) | 1  (Ref) | 1  (Ref) | 1  (Ref) |
| More than 18 years | 1.41  (0.85, 2.34) | 1.83  (1.16, 2.88) | 2.59  (1.10, 6.07) | 2.57  (1.48, 4.44) |
|  |  |  |  |  |
| **Start Time** |  |  |  |  |
| 07:30 | 1  (Ref) | 1  (Ref) | 1  (Ref) | 1  (Ref) |
| 10:00 | 0.85  (0.78, 0.92) | 0.92  (0.83, 1.01) | 0.99  (0.91, 1.08) | 0.87  (0.79, 0.95) |
| 12:30 | 0.71  (0.65, 0.76) | 0.96  (0.87, 1.06) | 0.73  (0.67, 0.80) | 0.77  (0.71, 0.85) |
| 15:00 | 0.73  (0.67, 0.79) | 0.76  (0.69, 0.84) | 0.78  (0.71, 0.85) | 0.78  (0.71, 0.86) |
| 17:30 | 0.74  (0.68, 0.80) | 0.86  (0.78, 0.94) | 0.81  (0.74, 0.88) | 0.97  (0.88, 1.06) |
|  |  |  |  |  |
| **Day of week** |  |  |  |  |
| Weekday | 1  (Ref) | 1  (Ref) | 1  (Ref) | 1  (Ref) |
| Weekend | 0.91  (0.86, 0.96) | 1.10  (1.03, 1.17) | 0.91  (0.82, 1.01) | 1.06  (0.96, 1.18) |
|  |  |  |  |  |
| **Location^a^** |  |  |  |  |
| Location 1 | 1  (Ref) | 1  (Ref) | 1  (Ref) | 1  (Ref) |
| Location 2 | 0.44  (0.40, 0.47) | 0.55  (0.51, 0.61) | 0.74  (0.67, 0.81) | 0.93  (0.84, 1.02) |
| Location 3 | 0.39  (0.36, 0.43) | 0.60  (0.55, 0.65) | 0.41  (0.38, 0.44) | 0.53  (0.49, 0.58) |
| Location 4 | 0.27  (0.25, 0.30) | 0.58  (0.53, 0.64) | 0.32  (0.29, 0.35) | 0.52  (0.46, 0.58) |
| Location 5 | 0.53  (0.49, 0.57) | 0.26  (0.24, 0.29) | 0.51  (0.47, 0.56) | 0.56  (0.51, 0.61) |

^a^Location 1: Eastern Express Highway, Vikhroli; Location 2: Jogeshwari Vikhroli Link Road (JVLR); Location 3: General Arun Kumar Vaidya (GAKV) Road; Location 4: Dadabhai Naoroji Road; Location 5: Netaji Subash Chandra Bose Road
