# Supplementary material for: Paper Versus Digital Data Collection Methods for Road Safety Observations: Comparative Efficiency Analysis of Cost, Timeliness, Reliability, and Results
Source: J Med Internet Res. 2020 May 22;22(5):e17129. doi: 10.2196/17129 (PMC7275261; doi:10.2196/17129)
Supplement: Multimedia Appendix 6 [file jmir_v22i5e17129_app6.docx]

Multimedia Appendix 6. Seatbelt use: adjusted odds ratios (aOR) and 95% confidence intervals (CI) by round and method of data collection.

|  | **Winter** | **Winter** | **Summer** | **Summer** |
| --- | --- | --- | --- | --- |
|  | Paper | Digital | Paper | Digital |
|  | aOR (95% CI) | aOR (95% CI) | aOR (95% CI) | aOR (95% CI) |
|  |  |  |  |  |
| **Occupant role** |  |  |  |  |
| Drivers | 1 (Ref) | 1 (Ref) | 1 (Ref) | 1 (Ref) |
| Front passenger | 0.041 (0.037, 0.044) | 0.055 (0.050, 0.061) | 0.032 (0.029, 0.035) | 0.043 (0.039, 0.048) |
| Rear passenger | 0.002 (0.002, 0.003) | 0.002 (0.001, 0.002) | 0.001 (0.001, 0.002) | 0.001 (0.001, 0.002) |
|  |  |  |  |  |
| **Occupant Sex** |  |  |  |  |
| Female | 1 (Ref) | 1 (Ref) | 1 (Ref) | 1 (Ref) |
| Male | 1.47 (1.31, 1.66) | 1.45 (1.26, 1.68) | 1.37 (1.23, 1.57) | 1.69 (1.46, 1.94) |
|  |  |  |  |  |
| **Occupant Age** |  |  |  |  |
| 12-17 years | 0.75 (0.45, 1.24) | 0.34 (0.18, 0.66) | 0.51 (0.30, 0.878) | 0.55 (0.32, 0.94) |
| 18-24 years | 0.50 (0.41, 0.62) | 0.66 (0.56, 0.78) | 0.57 (0.43, 0.75) | 0.62 (0.50, 0.77) |
| 25-59 years | 1 (Ref) | 1 (Ref) | 1 (Ref) | 1 (Ref) |
| More than 60 | 0.69 (0.51, 0.93) | 0.76 (0.55, 1.06) | 0.61 (0.49, 0.77) | 0.69 (0.51, 0.92) |
|  |  |  |  |  |
| **Start Time** |  |  |  |  |
| 7:30 | 1 (Ref) | 1 (Ref) | 1 (Ref) | 1 (Ref) |
| 10:00 | 1.06 (0.94, 1.20) | 1.12 (0.97, 1.29) | 1.29 (1.13, 1.48) | 1.52 (1.31, 1.76) |
| 12:30 | 0.99 (0.88, 1.12) | 1.04 (0.91, 1.20) | 1.14 (1.00, 1.30) | 1.56 (1.35, 1.81) |
| 15:00 | 0.95 (0.84, 1.07) | 0.85 (0.74, 0.98) | 1.18 (1.04, 1.34) | 1.50 (1.31, 1.73) |
| 17:30 | 1.10 (0.98, 1.24) | 1.09 (0.95, 1.25) | 1.24 (1.09, 1.40) | 1.81 (1.58, 2.08) |
|  |  |  |  |  |
| **Day of week** |  |  |  |  |
| Weekday | 1 (Ref) | 1 (Ref) | 1 (Ref) | 1 (Ref) |
| Weekend | 0.84 (0.77, 0.91) | 0.74 (0.66, 0.83) | 0.87 (0.79, 0.95) | 0.99 (0.90, 1.10) |
|  |  |  |  |  |
| **Location^a^** |  |  |  |  |
| Location 1 | 1 (Ref) | 1 (Ref) | 1 (Ref) | 1 (Ref) |
| Location 2 | 0.96 (0.86, 1.08) | 1.86 (1.61, 2.14) | 2.55 (2.21, 2.93) | 2.64 (2.26, 3.07) |
| Location 3 | 0.40 (0.35, 0.45) | 0.44 (0.38, 0.50) | 0.73 (0.65, 0.82) | 0.73 (0.64, 0.84) |
| Location 4 | 0.45 (0.40, 0.51) | 0.53 (0.46, 0.61) | 0.61 (0.54, 0.69) | 0.73 (0.64, 0.84) |
| Location 5 | 0.53 (0.46, 0.61) | 1.04 (0.87, 1.24) | 0.86 (0.76, 0.98) | 1.11 (0.96, 1.27) |
|  |  |  |  |  |
| **Vehicle Type** |  |  |  |  |
| Sedan/Saloon | 1 (Ref) | 1 (Ref) | 1 (Ref) | 1 (Ref) |
| Pickup/Light truck | 1.62 (0.95, 2.77) | 0.63 (0.50, 0.80) | 0.67 (0.38, 1.20) | 1.10 (0.72, 1.70) |
| Truck/Large truck | 0.11 (0.06, 0.18) | 0.04 (0.03, 0.05) | 0.07 (0.04, 0.13) | 0.10 (0.07, 0.16) |
| Bus | 0.014 (0.010, 0.018) | 0.009 (0.006, 0.01) | 0.007 (0.004, 0.01) | 0.01 (0.007, 0.017) |
| Minibus/Minivan | 0.15 (0.09, 0.24) | 0.16 (0.12, 0.22) | 0.08 (0.04, 0.14) | 0.23 (0.15, 0.37) |
| SUV/4WD | 0.84 (0.75, 0.93) | 0.85 (0.74, 0.97) | 0.85 (0.76, 0.95) | 0.85 (0.75, 0.97) |
|  |  |  |  |  |
| **Vehicle Ownership** |  |  |  |  |
| Private | 19.40 (14.24, 26.45) | 6.94 (4.93, 9.78) | 19.67 (12.78, 30.28) | 18.86 (12.75, 27.90) |
| Commercial | 6.58 (3.77, 11.50) | 5.56 (3.86, 8.02) | 13.30 (8.86, 19.97) | 7.06 (4.92, 10.14) |
| Government | 1 (Ref) | 1 (Ref) | 1 (Ref) | 1 (Ref) |
| Taxi | 13.59 (9.93, 18.61) | 5.12 (3.68, 7.35) | 13.95 (9.04, 21.50) | 11.20 (7.53, 16.64) |
| Tourist vehicle | 22.86 (11.94, 43.76) | 8.67 (3.72, 20.21) | 21.61 (10.05, 46.46) | 17.10 (9.08, 32.17) |
|  |  |  |  |  |
| **Weather** |  |  |  |  |
| Dry/no rain | N/A | N/A | 1 (Ref) | 1 (Ref) |
| Light rain / drizzle | N/A | N/A | 0.74 (0.62, 0.90) | 0.95 (0.77, 1.18) |

^a^Location 1: Eastern Express Highway, Vikhroli; Location 2: Jogeshwari Vikhroli Link Road (JVLR); Location 3: General Arun Kumar Vaidya (GAKV) Road; Location 4: Dadabhai Naoroji Road; Location 5: Netaji Subash Chandra Bose Road
