# Supplementary material for: Paper Versus Digital Data Collection Methods for Road Safety Observations: Comparative Efficiency Analysis of Cost, Timeliness, Reliability, and Results
Source: J Med Internet Res. 2020 May 22;22(5):e17129. doi: 10.2196/17129 (PMC7275261; doi:10.2196/17129)
Supplement: Multimedia Appendix 7 [file jmir_v22i5e17129_app7.docx]

Multimedia Appendix 7. Speeding: adjusted odds ratios (aOR) and 95% confidence intervals (CI) by round and method of data collection**.**

|  | **Winter** | **Winter** | **Summer** | **Summer** |
| --- | --- | --- | --- | --- |
|  | Paper | Digital | Paper | Digital |
|  | aOR (95% CI) | aOR (95% CI) | aOR (95% CI) | aOR (95% CI) |
|  |  |  |  |  |
| **Start Time** |  |  |  |  |
| 7:30 | 1 (Ref) | 1 (Ref) | 1 (Ref) | 1 (Ref) |
| 10:00 | 0.81 (0.70, 0.92) | 0.92 (0.80, 1.06) | 0.58 (0.51, 0.66) | 0.92 (0.81, 1.04) |
| 12:30 | 0.68 (0.59, 0.79) | 0.86 (0.74, 0.99) | 0.69 (0.61, 0.78) | 0.96 (0.85, 1.09) |
| 15:00 | 0.73 (0.64, 0.84) | 0.55 (0.47, 0.64) | 0.65 (0.57, 0.74) | 0.80 (0.71, 0.92) |
| 17:30 | 0.55 (0.48, 0.64) | 0.52 (0.44, 0.60) | 0.34 (0.29, 0.39) | 0.78 (0.68, 0.89) |
|  |  |  |  |  |
| **Day of week** |  |  |  |  |
| Weekday | 1 (Ref) | 1 (Ref) | 1 (Ref) | 1 (Ref) |
| Weekend | 0.87 (0.79, 0.96) | 0.86 (0.77, 0.96) | 2.61 (2.39, 2.86) | 2.72 (2.50, 2.96) |
|  |  |  |  |  |
| **Vehicle Type** |  |  |  |  |
| Motorcycle | 1 (Ref) | 1 (Ref) | 1 (Ref) | 1 (Ref) |
| Sedan/Saloon | 0.24 (0.22, 0.27) | 0.27 (0.24, 0.31) | 0.62 (0.55, 0.69) | 0.48 (0.44, 0.53) |
| Pickup/Light truck | 0.33 (0.06, 1.86) | 0.09 (0.06, 0.15) | 0.18 (0.07, 0.47) | 0.21 (0.12, 0.38) |
| Truck/Large truck | 0.25 (0.05, 1.32) | 0.04 (0.02, 0.08) | 0.17 (0.07, 0.45) | 0.20 (0.11, 0.37) |
| Bus | 0.12 (0.07, 0.21) | 0.04 (0.02, 0.10) | 0.14 (0.06, 0.34) | 0.18 (0.10, 0.33) |
| Minibus/Minivan | 0.15 (0.02, 1.03) | 0.14 (0.06, 0.32) | 0.28 (0.10, 0.79) | 0.22 (0.10, 0.50) |
| SUV/4WD | 0.28 (0.24, 0.33) | 0.34 (0.29, 0.40) | 0.58 (0.50, 0.67) | 0.51 (0.45, 0.59) |
|  |  |  |  |  |
| **Vehicle Ownership** |  |  |  |  |
| Private | 1 (Ref) | 1 (Ref) | 1 (Ref) | 1 (Ref) |
| Commercial | 0.24 (0.04, 1.26) | 0.67 (0.52, 0.86) | 1.84 (0.72, 4.74) | 1.06 (0.60, 1.87) |
| Government | 0.27 (0.11, 0.70) | 0.64 (0.34, 1.22) | 0.71 (0.33, 1.52) | 0.43 (0.23, 0.79) |
| Taxi | 0.42 (0.35, 0.49) | 0.43 (0.35 0.52) | 0.65 (0.58, 0.74) | 0.65 (0.58, 0.73) |
| Tourist vehicle | 0.71 (0.20, 2.59) | 0.83 (0.25, 2.75) | 2.54 (0.59, 10.95) | 1.96 (0.98, 3.95) |
|  |  |  |  |  |
| **Speed Deterrents** |  |  |  |  |
| No deterrents | 1 (Ref) | 1 (Ref) | N/A | N/A |
| Stop sign, only | 0.33 (0.28, 0.38) | 0.34 (0.30, 0.39) | N/A | N/A |
| All deterrents | 0.005 (0.002, 0.012) | 0.018 (0.012, 0.028) | N/A | N/A |
